# Examiner effect on the objective structured clinical exam – a study at five medical schools

**DOI:** 10.1186/s12909-017-0908-1

**Published:** 2017-04-24

**Authors:** Iris Schleicher, Karsten Leitner, Jana Juenger, Andreas Moeltner, Miriam Ruesseler, Bernd Bender, Jasmina Sterz, Karl-Friedrich Schuettler, Sarah Koenig, Joachim Gerhard Kreuder

**Affiliations:** 1 Department of Orthopaedics, Trauma Surgery and Sportsmedicine, Agaplesion ev. Hospital Giessen, Paul-Zipp-Str.171, 35398 Giessen, Germany; 2 Department of Psychosomatic and General Internal Medicine, University of Heidelberg, 69120 Heidelberg, Germany; 3 Center of Excellence in Medical Assessment, Faculty of Medicine, University of Heidelberg, im Neuenheimer Feld 346, 69120 Heidelberg, Germany; 4 Department of Trauma, Hand and Reconstructive Surgery, University of Frankfurt, Theodor Stern Kai 7, 60590 Frankfurt am Main, Germany; 5 Department of General Surgery, University of Frankfurt, Theodor Stern Kai, 60590 Frankfurt am Main, Germany; 6 Center for Orthopaedics and Trauma Surgery, University Hospital Giessen and Marburg GmbH, Location Marburg, 35043 Marburg, Germany; 7 Department of General Surgery, University of Goettingen, Robert-Koch-Straße 40, 37075 Göttingen, Germany; 8 Present address: Institute for medical and pharmaceutical tests, Große Langgasse 8, 55116 Mainz, Germany; 9 Present address: Institute for medical education and educational research, Josef-Schneider-Str. 2/D6, 97080 Würzburg, Germany

**Keywords:** OSCE, Bias, Assessment, Practical skills, Medical student

## Abstract

**Background:**

The Objective Structured Clinical Examination (OSCE) is increasingly used at medical schools to assess practical competencies. To compare the outcomes of students at different medical schools, we introduced standardized OSCE stations with identical checklists.

**Methods:**

We investigated examiner bias at standardized OSCE stations for knee- and shoulder-joint examinations, which were implemented into the surgical OSCE at five different medical schools. The checklists for the assessment consisted of part A for knowledge and performance of the skill and part B for communication and interaction with the patient. At each medical faculty, one reference examiner also scored independently to the local examiner. The scores from both examiners were compared and analysed for inter-rater reliability and correlation with the level of clinical experience. Possible gender bias was also evaluated.

**Results:**

In part A of the checklist, local examiners graded students higher compared to the reference examiner; in part B of the checklist, there was no trend to the findings. The inter-rater reliability was weak, and the scoring correlated only weakly with the examiner’s level of experience. Female examiners rated generally higher, but male examiners scored significantly higher if the examinee was female.

**Conclusions:**

These findings of examiner effects, even in standardized situations, may influence outcome even when students perform equally well. Examiners need to be made aware of these biases prior to examining.

**Electronic supplementary material:**

The online version of this article (doi:10.1186/s12909-017-0908-1) contains supplementary material, which is available to authorized users.

## Background

Objective Structured Clinical Examination (OSCE) is widely used to assess practical skills during medical studies [1, 2]. Countries like the United States of America, Canada, and Switzerland have already introduced standardized OSCE into their exams in order to evaluate clinical and practical competencies [3, 4], whereas in Germany, the organization, content, and grading of an OSCE is completely regulated by each medical faculty. Thus, there are difficulties when trying to compare outcomes or standards of medical students from different faculties. A lot of work is needed for the preparation of these exams. Further, different medical faculties need to agree on the content, standards, and benchmarks for OSCEs, as experiences from Switzerland demonstrate [3]. Networks like the Umbrella Consortium for Assessment Networks (UCAN), which aids in the cooperation and sharing of resources such as exams and assessments, were only recently founded.

Despite the increasing introduction of OSCE to assess clinical competencies, there are concerns of higher variability [5]. Although studies demonstrate high reliability for OSCE among different sites and languages [6], other studies report variability in the content, checklists, and outcomes as well as a high examiner-dependent effect [7–10]. McManus et al. identified the bias of the examiner as having a meaningful influence on the candidates’ outcome. Further, their study discussed the effect of the stringency-leniency-effect, which is also known as the “hawk-dove-effect” [9]. Iramaneerat et al. described four issues that contribute to the rater-effect: leniency, inconsistency, the “halo-effect”, and a limited range of scores of the examiner [11]. The gender of the examiner as a possible factor of bias was evaluated in other studies. In some studies, female examiners tend to score higher [12, 13], whereas many other studies could not confirm a gender-related effect [14–16]. In contrast, other studies found that male examiners score female students higher [17].

The aim of this study was to implement standardized OSCE stations at five different medical schools in order to get a tool to compare the outcomes of practical skill testing. In addition to assessing student performance, we studied the contribution of the examiner. A reference examiner and a local examiner scored simultaneously each candidate. In order to evaluate the effect of the examiner, the three main research questions for this study were:

1. Is there a difference between the scoring of the reference examiner and the local examiners?
2. Does the amount of the examiner’s clinical experience influence scoring?
3. Is the scoring biased by the examiner’s and student’s gender?

## Methods

Learning objectives for joint examinations are included in the National Catalogue of Learning Objectives in Medicine [18], which was approved by each medical faculty in Germany. Beforehand, each medical school had individual surgical OSCE-stations (for example assessing knee-joint examination by testing only ligament stability tests), thus we created basic and consistent OSCE-stations, which implemented a complete structured knee- or shoulder-joint examination.

Checklists to assess structure, performance, and knowledge of the joint examination were developed and students were scored using a 3-step-Likert-scale (part A) (Additional files 1 and 2). Additionally, how well the student communicated and interacted with the patient was scored using a global rating scale (part B) (Additional file 3) with 5 items, each being scored on a 5-step-scale. Part B of the checklist was equal for both joint assessments. Scores from each joint examination were then tallied in a way that two-thirds and one-third of a student’s score were from part A and part B, respectively.

Students had up to 5 min to perform and explain the joint examination to a standardized patient, an actor or actress who had been instructed to play a patient in a standardized, consistent role (for example a patient with typical impingement syndrome of the shoulder).

Five German medical schools (named in the following sections sites (S) 1–5) agreed to implement the standardized OSCE-stations in their local surgical OSCE.

To minimize bias from different central examiners, we appointed a single reference examiner to assess each student in addition to a local examiner. The reference examiner was a male resident of orthopaedic surgery with long experience in assessing practical skills during OSCE for which he had completed several rater trainings beforehand. He scored every student with the original checklist and his results were later used for comparison of outcome at the different medical schools.

For this study, outcome of the basic, consistent part of the checklists were evaluated, and the scores from the reference examiner were compared to the ones from the local examiner to calculate interrater-reliability. Because local exams are a matter of each medical faculty themselves, each medical school could add items for their local outcome, for example about further diagnostic investigation (Ultrasound, X-ray, MRI). However, it was not allowed to omit a basic item. Also some medical schools used their own raw scoring system in order to stick to the scoring points of other OSCE-station (for example all scores were doubled). By comparing in percentage points it was possible to compare different sites even if the raw scoring was different as long as the items were all scored separately or the grouping in rubrics was comparable.

Depending on the medical faculty, between two and four local examiners with different levels of professional (clinical) experience administered the OSCE. Thus, results were correlated with the examiner’s level of clinical experience and evaluated in relation to their gender.

Altogether, 180, 147, 137, 31, and 45 students from sites 1, 2, 3, 4, and 5, respectively, were included in the study. Unfortunately, the local checklists of site 4 differed to the original, standardized checklists; thus, only the scores of the reference examiner (by using the original checklists) were used for evaluation. Although including all the agreed items, items at site 4 were not scored separately and the 3-step Likert-scale was not used. Part B of the local checklist at site 3 was excluded because some agreed items were not scored separately.

The study was approved by the ethics committee of the organizing university.

### Statistics

Because the reference examiner and one local rater assessed every student, the means and standard deviation of both ratings were calculated and compared. Additionally, results were calculated separately for male and female examiners. Significant mean differences were evaluated with Analysis of variance (ANOVA) if distribution was normal or Kruskal-Wallis test if not. Significant differences between individual sites were identified by comparing pairs using the Duncan test. Differences were considered significant if *p* < 0.05. Interrater-reliability was calculated and expressed using the Kendall-W coefficient. The Kendall-Tau-b coefficient was applied in order to evaluate correlation between the examiner’s level of clinical experience and the student’s outcome. For expressing effect strength for significant differences in the gender analyses, Cohen’s coefficient d was calculated. IBM SPSS version 19 (SPSS, Inc., Chicago, IL, USA) was used for the statistical analyses.

## Results

### Comparison of reference and local examiners

**Fig. 1:**
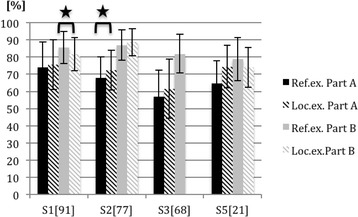
Mean outcomes and standard deviations of part A and B of the knee examinations from different sites (S); comparison of scores awarded by the reference examiner (Ref.ex.) and the local examiner (Loc.ex.). Number of students per site is in brackets. Significant differences between the scoring of the reference and local examiners are marked with a horizontal bracket and a star

**Fig. 2:**
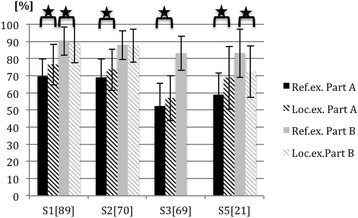
Mean outcomes and standard deviations for part A and B of the shoulder examination from different sites (S); comparison of scores awarded by the reference examiner (Ref.ex.) and local examiners (Loc.ex.). Number of students per site is in brackets. Significant differences between the scoring of reference and local examiners are marked with a horizontal bracket and a star

Results from part A of the checklist for both joint examinations indicated that at all sites tested a higher score was given by the local examiners than by the reference examiner. The differences at part A were significant for all included sites for the shoulder joint examination, but only at site 2 for the knee examination. Because of too many differences regarding the scoring of items and their grouping in rubrics in their local checklist, site 4 could not be included in this statistical evaluation. Also, part B of the local checklist at site 3 could not be compared to the reference checklist; thus, part B of the checklist was only comparable at site 1, 2, and 5. Significant differences between the scoring of the reference and local examiner at part B were seen at site 1 for both joint examinations (*p* = 0.025 for knee, *p* = 0.003 for shoulder) and at site 5 for the shoulder examination (*p* = 0.022). Unlike part A, scores of the local examiner for parts B were not consistently higher when compared to the scores given by the reference examiner. The mean results and standard deviations of the reference and local examiners’ scoring of both joint assessments are illustrated in Figs. 1 and 2.

The Kendall-W-concordance coefficient gives values between 0 (no concordance) and 1 (complete consensus). In this study, it was calculated between 0.158 and 0.387 for interrater-reliability, which means there was only low agreement between the reference and local examiners (see Table 1).

**Table 1:** Interrater-reliability (Kendall-W coefficient) between the reference and local examiners at different sites (S) for part A of the shoulder and knee examinations and part B (because part B was identical for both examinations, one value is given)

	Kendall-W coefficient of concordance		
	Shoulder exam Part A	Knee exam Part A	Part B
S1	0.24	0.158	0.348
S2	0.248	0.247	0.256
S3	0.314	0.292	-
S5	0.215	0.387	0.25

### Level of experience

**Fig. 3:**
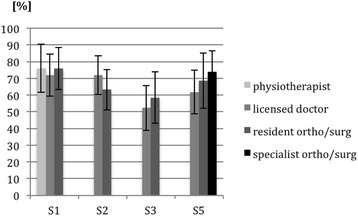
Mean outcomes and standard deviations (in percentages) for part A of the knee and shoulder examinations from different sites (S). The data are separated by the level of professional clinical experience of the examiner (physiotherapist, licensed doctor without clinical experience, resident in general surgery/orthopaedic surgery, and specialist in general surgery/orthopaedic surgery

**Fig. 4:**
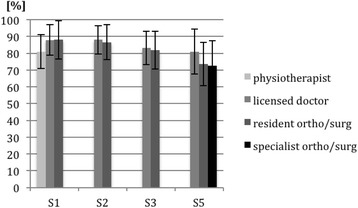
Mean outcomes and standard deviations (in percentages) for part B of the knee and shoulder examinations from different sites (S). Data are separated by the level of professional clinical experience of the examiner (physiotherapist, licensed doctor without clinical experience, resident in general surgery/orthopaedic surgery, and specialist in general surgery/orthopaedic surgery

Most examiners were licensed (and not yet involved in their residency) or residents of a surgical or orthopaedic surgery department. At site 1, one of the local examiners was a physiotherapist; and at site 5, one rater was an orthopaedic surgery specialist with many years of clinical experience. Scoring was unaffected by the level of the examiner’s clinical experience (see Figs. 3 and 4).

The correlation between the examiners’ clinical experience level and scoring was calculated using the Kendall-Tau-b coefficient. Values between 0 (no correlation) and 1(complete correlation) were obtained. Although the correlations were mainly significant, the coefficients had changing positive and negative signs, which signified weak correlations (Table 2).

**Table 2:** Correlations between the mean scores of examiners with different levels of clinical experience (physiotherapists, licensed doctors without clinical experience, residents in general surgery/orthopaedic surgery, and specialist in general surgery/orthopaedic surgery) for part A and B of the shoulder/knee examinations at different sites (S)

	Part A		Part B	
	Correlation (Kendall-Tau-b coefficients)	*p* values	Correlation (Kendall-Tau-b coefficients)	*p* values
S1	0.129	0.002	−0.127	0.003
S2	-0.208	<0.001	−0.034	ns
S3	0.15	0.003	-	-
S5	0.293	0.001	−0.299	0.001

Differences were considered significant if *p* < 0.05; “ns” means non-significant. Correlations are expressed as Kendall-Tau-b coefficients

### Gender

The proportion of female students was higher than that of males except at S4 (S1: 114 females/66 males, S2: 79 females/68 males, S3: 89 females/48 males, S4 12 females/19 males, and S5: 27 females/15 males). Regarding the gender of examiners that could be included in the study, 837 and 206 of the examinations were performed by a male and female examiner, respectively.

**Fig. 5:**
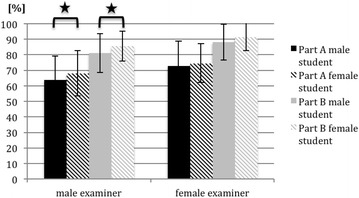
Mean outcomes and standard deviations for part A and B of the joint examinations of all participating medical schools. Data are separated by the gender of examiner and examinee (student). Significant differences are marked with a horizontal bracket and a star

At all sites, female students were scored higher on both parts of the checklists and female examiners generally awarded higher scores. Nevertheless, a significant finding was that male examiners gave higher scores to female examinees for both part A (*p* = 0.025) and part B (*p* = 0.04) of the checklist (see Fig. 5). Cohen’s coefficient expresses the effect strength. If d is >0.2 to 0.4 it signifies a weak effect, >0.4 to 0.7 a moderate effect and 0.8 and higher a strong effect. Cohen’s d coefficient for effect strength of gender bias was weak (d = 0.29 and d = 0.32 for part A and part B, respectively).

## Discussion

In order to create a tool for outcome-orientated comparison of practical and clinical competencies between different medical schools, it is important to agree upon standards, content, and method of assessment. Therefore, we developed standardized OSCE-stations with checklists. These exams were implemented into the surgical OSCE of five different medical schools. Students who took the OSCE were scored by both the reference examiner and one local examiner. Extensive analyses of the outcomes between participating faculties are described in a different article (not yet published). The aim of this work was to evaluate the impact of the examiner as a factor that could affect OSCE scores. This is important if the OSCE is to be used by multiple medical faculties to accurately and fairly assess the competency of students. To our knowledge, only one other study has evaluated the outcome of a standardized OSCE administered in different faculties [7]. In contrast to our study in which students were scored by the same reference examiner and a local rater, several central examiners were appointed. Significant variations in the scores between the participating medical schools and also between the central and local examiners were detected [7]. In our study that utilized a single reference or central examiner, significant differences were observed in the scores given by the reference and those given by the local examiner; interrater-reliability was low. Many authors describe examiner bias when reporting clinical exam results. Mostly, the “hawk-dove” effect is mentioned, which means that some examiners are consistently stringent, while others are consistently lenient. This effect is observed in many studies [9, 10, 19] and cannot be easily eliminated. Some authors conclude that the stringency is a part of the examiner’s personality and that the outcome of the exam is more predictable if this is not changed [9]. Instead, it is recommended that tests are scored by a pair of examiners [13]. In our study, we used a pair of examiners. However, they were not allowed to discuss scoring so that we could get valid data regarding the interrater-reliability. Our finding of low interrater-reliability was described by other authors [20]. In contrast, others describe high interrater-reliability but question the validity of the scores [21]. In our study, even when using checklists that are easily filled in, the fact that for the same performance mean scores given by the reference and the local examiners differed as much as 10–15% at one medical school in both checklists of the shoulder examination and at checklist A of the knee examination at another site is definitely disconcerting. Although the different weighing of checklist A and B for the total score did not result in a total difference of 10% between reference and local examiner, it underlines the imperative for regular evaluation and training of examiners.

Similar to other authors, we could not correlate scores given by the examiners to their level of clinical experience [22]. Clinical experience does not necessarily imply being a consistent and fair examiner. The reference examiner was on a rather low level of clinical experience but well trained in OSCE as a form of testing, but not necessarily made aware of the potential for examiner bias. Nevertheless, he had a lot of experience as a proctor and examiner, and this might be the reason that his scoring was more stringent. Many other authors observed a similar finding with increasing numbers of examinations [9, 23]. Two British studies evaluated the effect of examiner bias on OSCE for assessing communication skills, each at one faculty. One study did not see an effect when the number of examinations was increased. However, they did observe that raters were more inconsistent at the end of an assessment period [22]. Another study could not confirm that examiners’ fatigue was related to the duration of the OSCE [24]. In our study we assumed that the reference examiner was consistent during the examinations. Though overall stringent, no change in stringency was observed during examinations. However, inconsistency in rating can only be kept to a minimum by training and evaluating but never be ruled out completely.

One important bias that we observed in our study was related to gender. Even though the effect was weak, male examiners at all faculties scored female candidates significantly higher. This raises the question of if male examiners are more lenient on female examinees? The findings of other studies investigating gender bias are not consistent. Boehm et al. observed the same effect of male examiners rating female examinees higher. We also found that female examiners generally give higher scores than male examiners, which confirms the findings of other authors [12]. However, the majority of studies could not detect a gender bias at all [13, 15, 16]. In a retrospective study, one group even described how female examiners gave lower scores regardless of the gender of the examinee [25].

Gender or age of the standardized patient is a further possible source of bias, especially when involved in scoring. Because the standardized patients did not contribute to the scores of our OSCE, we did not analyse this effect; other studies could not detect a bias [12, 14].

McManus et al. contributed the examiner variance as 12% of the systematic variance. In that study, 1% of the variance depended on the differences in difficulties of the station and 87% on the differences of the candidates [9]. Rater training can help to improve examiner’s variance in scoring, although Weitz et al. did not observe a measurable influence on the accuracy of testing by increasing rater training [26]. Nevertheless, examiners should be made aware of potential effects and biases; regular reviews of clinical and practical exams are recommended [27]. Over all, OSCE are shown to have many good effects on students, curriculum, and faculty development [28]. In addition to rating the performance of students, the goal of the assessment should be also to motivate students, while being aware of the bias [29].

The inconsistency of local checklists especially at one site was a limitation of the study. This raises also the question how far an examination can be standardized. The two stations, the examination itself and agreed items were consistent, but differences in detailed scoring of the items produced difficulties to compare results of reference and local examiner, even when applying percentage points instead of raw points. For comparison of students’ outcome at the five medical schools (which is discussed in a further work) this is sufficient as only the scores of the reference examiner who used these original checklists contributed. Nevertheless, even by comparing scores of different examiners only at 4 sites, examiner effects were observed.

## Conclusion

Altogether, we could confirm a gender-related bias in different medical schools. Further, we identified a low conformity of scores between different examiners, which is concerning. This variability may introduce errors into ratings, which are independent of the student’s performance. Through training, examiners should be made aware of potential bias, for example by implementation of methods like role-playing. This might have positive influence on examiner bias and should be further investigated in order to get fair results during exams.

## Additional files

Additional file 1: Part A: Checklist “examination of knee-joint“. Description of data: Blank English version of checklist part A with a 3-step-Likert-scale for OSCE station “knee-joint examination”. (DOC 38 kb)

Additional file 2: Part A: Checklist “examination of shoulder-joint“. Blank English version of checklist part A with a 3-step-Likert-scale for OSCE station “shoulder-joint examination”. (DOC 45 kb)

Additional file 3: Part B: Checklist for communication and interaction. Blank English version of checklist part B with a global rating scale including 5 items, each being scored on a 5-step-scale for OSCE stations testing joint examination. (DOC 36 kb)

## Abbreviations

ANOVA: Analyses of variance
OSCE: Objective structured clinical examination
S: Sites
UCAN: Umbrella consortium for assessment networks

## References

[1] Khan KZ, Ramachandran S, Gaunt K, Pushkar P (2013). The Objective Structured Clinical Examination (OSCE): AMEE Guide No. 81. Part I: an historical and theoretical perspective. Med Teach.

[2] Harden RM, Stevenson M, Downie WW, Wilson GM (1975). Assessment of clinical competence using objective structured examination. Br Med J.

[3] Guttormsen S, Beyeler C, Bonvin R, Feller S, Schirlo C, Schnabel K, Schurter T, Berendonk C (2013). The new licencing examination for human medicine: from concept to implementation. Swiss Med Wkly.

[4] De Champlain A, Swygert K, Swanson DB, Boulet JR (2006). Assessing the underlying structure of the United States Medical Licensing Examination Step 2 test of clinical skills using confirmatory factor analysis. Acad Med.

[5] Turner JL, Dankoski ME (2008). Objective structured clinical exams: a critical review. Fam Med.

[6] Brailovsky CA, Grand'Maison P, Lescop J (1992). A large-scale multicenter objective structured clinical examination for licensure. Acad Med.

[7] Chesser A, Cameron H, Evans P, Cleland J, Boursicot K, Mires G (2009). Sources of variation in performance on a shared OSCE station across four UK medical schools. Med Educ.

[8] Makinen M, Axelsson A, Castren M, Nurmi J, Lankinen I, Niemi-Murola L (2010). Assessment of CPR-D skills of nursing students in two institutions: reality versus recommendations in the guidelines. Eur. J. Emerg. Med.

[9] McManus IC, Thompson M, Mollon J (2006). Assessment of examiner leniency and stringency ('hawk-dove effect') in the MRCP(UK) clinical examination (PACES) using multi-facet Rasch modelling. BMC Med Educ.

[10] Harasym PH, Woloschuk W, Cunning L (2008). Undesired variance due to examiner stringency/leniency effect in communication skill scores assessed in OSCEs. Adv Health Sci Educ Theory Pract.

[11] Iramaneerat C, Yudkowsky R (2007). Rater errors in a clinical skills assessment of medical students. Eval Health Prof.

[12] Wiskin CM, Allan TF, Skelton JR (2004). Gender as a variable in the assessment of final year degree-level communication skills. Med Educ.

[13] McManus IC, Elder AT, Dacre J (2013). Investigating possible ethnicity and sex bias in clinical examiners: an analysis of data from the MRCP(UK) PACES and nPACES examinations. BMC Med Educ.

[14] Colliver JA, Vu NV, Marcy ML, Travis TA, Robbs RS (1993). Effects of examinee gender, standardized-patient gender, and their interaction on standardized patients' ratings of examinees' interpersonal and communication skills. Acad Med.

[15] Solomon DJ, Speer AJ, Ainsworth MA, DiPette DJ (1993). Investigating gender bias in preceptors' ratings of medical students. Acad Med.

[16] Denney ML, Freeman A, Wakeford R (2013). MRCGP CSA: are the examiners biased, favouring their own by sex, ethnicity, and degree source?. Br J Gen Pract.

[17] Boehm G BG, Kwizda-Gredler B, Kunze U, Rathmanner T, Rieder A, Schoberberger R, Schwarz B, Vutuc C, Kunze M. Einfluss von Geschlecht und Studiengebühren auf die Noten bei Rigorosum-Prüfungen im Prüfungsfach Sozialmedizin. In. Wien; 2001. http://didaktik-on.net/cgi-bin/didaktik.cgi?id=0000053.

[18] GMA. Nationaler kompetenzbasierter Lernzielkatalog in der Medizin (NKLM), Medizinischer Fakultätentag e.V. Gessellschaft für medizinische Ausbildung. In*.*, 1.07.2015 edn; 2015.

[19] Finn Y, Cantillon P, Flaherty G (2014). Exploration of a possible relationship between examiner stringency and personality factors in clinical assessments: a pilot study. BMC Med Educ.

[20] Kalet A, Earp JA, Kowlowitz V (1992). How well do faculty evaluate the interviewing skills of medical students?. J Gen Intern Med.

[21] Goldstein SD, Lindeman B, Colbert-Getz J, Arbella T, Dudas R, Lidor A, Sacks B (2014). Faculty and resident evaluations of medical students on a surgery clerkship correlate poorly with standardized exam scores. Am J Surg.

[22] Wiskin CM, Allan TF, Skelton JR (2003). Hitting the mark: negotiated marking and performance factors in the communication skills element of the VOICE examination. Med Educ.

[23] Hope D, Cameron H (2015). Examiners are most lenient at the start of a two-day OSCE. Med Teach.

[24] Humphris GM, Kaney S (2001). Examiner fatigue in communication skills objective structured clinical examinations. Med Educ.

[25] Grasl MC, Seemann R, Hanisch M, Heiduschka G, Kremser K, Thurnher D (2015). Influence of a revision course and the gender of examiners on the grades of the final ENT exam - a retrospective review of 3961 exams. GMS Z. Med. Ausbild.

[26] Weitz G, Vinzentius C, Twesten C, Lehnert H, Bonnemeier H, Konig IR (2014). Effects of a rater training on rating accuracy in a physical examination skills assessment. GMS Z. Med. Ausbild.

[27] Gispert R, Rue M, Roma J, Martinez-Carretero JM (1999). Gender, sequence of cases and day effects on clinical skills assessment with standardized patients. Med Educ.

[28] Duerson MC, Romrell LJ, Stevens CB (2000). Impacting faculty teaching and student performance: nine years' experience with the Objective Structured Clinical Examination. Teach Learn Med.

[29] Krupat E, Dienstag JL (2009). Commentary: Assessment is an educational tool. Acad Med.

