# Supplementary material for: Examiner effect on the objective structured clinical exam – a study at five medical schools
Source: BMC Med Educ. 2017 Apr 24;17:71. doi: 10.1186/s12909-017-0908-1 (PMC5402669; doi:10.1186/s12909-017-0908-1)
Supplement: Supplementary file 1 — Part A: Checklist “examination of knee-joint“. Description of data: Blank English version of checklist part A with a 3-step-Likert-scale for OSCE station “knee-joint examination”. (DOC 38 kb) [file 12909_2017_908_MOESM1_ESM.doc]

**Part A: Checklist „examination of knee-joint“**

| Examination | Failure to perform  0 | Partially/ incorrectly performed  1 | Correctly  performed  2 |
| --- | --- | --- | --- |
| Starts with examination of opposite side and compares to affected side. |  |  |  |
| **Inspection**: |  |  |  |
| Swelling, signs of trauma, redness, symmetry (for full scoring a minimum of 3 points must be mentioned) |  |  |  |
| Alignment of leg |  |  |  |
| **Palpation**: |  |  |  |
| Tender points (joint line, insertion of medial/lateral collateral ligament, insertion of patella tendon) (for full scoring a minimum of 2 points must be mentioned) |  |  |  |
| Dancing patella |  |  |  |
| Testing of peripheral neurovascular function |  |  |  |
| **Range of motion**: |  |  |  |
| Extension/flexion |  |  |  |
| **Tests for function**: |  |  |  |
| ACL-stability |  |  |  |
| PCL-Stability |  |  |  |
| Stability of collateral ligaments |  |  |  |
| Meniscal-signs |  |  |  |
| **Diagnostic tools**: |  |  |  |
| X-ray in 2 planes, MRI (for full scoring both points must be mentioned) |  |  |  |
