# Supplementary material for: Examiner effect on the objective structured clinical exam – a study at five medical schools
Source: BMC Med Educ. 2017 Apr 24;17:71. doi: 10.1186/s12909-017-0908-1 (PMC5402669; doi:10.1186/s12909-017-0908-1)
Supplement: Supplementary file 2 — Part A: Checklist “examination of shoulder-joint“. Blank English version of checklist part A with a 3-step-Likert-scale for OSCE station “shoulder-joint examination”. (DOC 45 kb) [file 12909_2017_908_MOESM2_ESM.doc]

**Part A: Checklist „examination of shoulder-joint“**

| Examination | Failure to perform  0 | Partially/ incorrectly performed  1 | Correctly  performed  2 |
| --- | --- | --- | --- |
| Starts with examination of opposite side and compares to affected side. |  |  |  |
| **Inspection**: (for full scoring a minimum of 2 points must be mentioned) |  |  |  |
| Swelling, relieving posture | **** | **** | **** |
| signs of trauma, colour changes, scars | **** | **** | **** |
| Symmetry of muscles, deformity | **** | **** | **** |
| **Palpation**: |  |  |  |
| Tenderpoints: AC-joint, SC-joint | **** | **** | **** |
| Greater and lesser tuberosity | **** | **** | **** |
| Bicipital groove, coracoid process | **** | **** | **** |
| Testing of peripheral neurovascular function | **** | **** | **** |
| **Range of motion:** |  |  |  |
| Abduction/Adduction | **** | **** | **** |
| Internal/external Rotation | **** | **** | **** |
| Anteversion/Retroversion | **** | **** | **** |
| Active *and* passive motion | **** | **** | **** |
| Neck-grip (combination of abduction/ext.rotation) | **** | **** | **** |
| Apron-grip (combination retroversion/int.rotation) | **** | **** | **** |
| **Functional tests:** |  |  |  |
| Painful arc | **** | **** | **** |
| Test for supraspinatus tendon | **** | **** | **** |
| Test for infraspinatus tendon | **** | **** | **** |
| Test for subscapularis tendon | **** | **** | **** |
| **Diagnostic tools:** |  |  |  |
| X-ray, 2 planes | **** | **** | **** |
