# Supplementary material for: Examiner effect on the objective structured clinical exam – a study at five medical schools
Source: BMC Med Educ. 2017 Apr 24;17:71. doi: 10.1186/s12909-017-0908-1 (PMC5402669; doi:10.1186/s12909-017-0908-1)
Supplement: Supplementary file 3 — Part B: Checklist for communication and interaction. Blank English version of checklist part B with a global rating scale including 5 items, each being scored on a 5-step-scale for OSCE stations testing joint examination. (DOC 36 kb) [file 12909_2017_908_MOESM3_ESM.doc]

**Part B: Checklist for communication and interaction**

|  | **1**  *failure* | **2** | **3**  *sufficient* | **4** | **5**  *excellent* |
| --- | --- | --- | --- | --- | --- |
| Introduction | Student does not mention own name |  | Student mentions own name without function and purpose |  | Student introduces him-/herself with name, function and purpose |
| Communication  /explanation of examination | Student does not communicate with patient |  | Student explains examination by using medical terms |  | Student explains examination in a generally understandable way |
| Sequence of examination | Completely unstructured, random order |  | Basic structure with some omissions or insertions |  | Follows sequence of checklist |
| Appearance | Student is unconfident, influent language, avoids eye-contact, speaks only with examiner |  | Student speaks with examiner and patient, nervous |  | Student speaks with patient, seems confident, fluent communication in adequate volume |
| Interaction | Student is unfriendly, not facing patient, interrupting patient |  | Student is mostly polite, sometimes interrupting patient or losing eye-contact |  | Student is polite, friendly, keeps eye-contact, does not interrupt patient and is responsive to questions of patient |
| Preconditions | Student does not pay attention to preconditions |  | Student cares partially for preconditions |  | Student cares for preconditions (privacy of patient, light, windows…) |
